# A New Noise-Tolerant Obstacle Avoidance Scheme for Motion Planning of Redundant Robot Manipulators

**DOI:** 10.3389/fnbot.2018.00051

**Published:** 2018-08-29

**Authors:** Dongsheng Guo, Feng Xu, Laicheng Yan, Zhuoyun Nie, Hui Shao

**Affiliations:** College of Information Science and Engineering, Huaqiao University, Xiamen, China

**Keywords:** obstacle avoidance, noise tolerant, pseudoinverse-type formulation, redundant robot manipulators, motion planning

## Abstract

Avoiding obstacle(s) is a challenging issue in the research of redundant robot manipulators. In addition, noise from truncation, rounding, and model uncertainty is an important factor that affects greatly the obstacle avoidance scheme. In this paper, based on the neural dynamics design formula, a new scheme with the pseudoinverse-type formulation is proposed for obstacle avoidance of redundant robot manipulators in a noisy environment. Such a scheme has the capability of suppressing constant and bounded time-varying noises, and it is thus termed as the noise-tolerant obstacle avoidance (NTOA) scheme in this paper. Theoretical results are also given to show the excellent property of the proposed NTOA scheme (particularly in noise situation). Based on a PA10 robot manipulator with point and window-shaped obstacles, computer simulation results are presented to further substantiate the efficacy and superiority of the proposed NTOA scheme for motion planning of redundant robot manipulators.

## 1. Introduction

Recently, redundant robot manipulators have played an increasingly important part in many scientific and industrial fields. Motion planning is the fundamental issue, and has been extensively studied (Siciliano and Khatib, 2008; Siciliano et al., 2009; Flacco and De luca, 2015; Qiu et al., 2016; Zhang et al., 2016; Li M. et al., 2017; Jin and Li, 2018). A collision-free motion is necessary for a redundant robot manipulator because collision would lead to the failure of the motion planning task. Moreover, such collision(s) may cause considerable damage to the robot manipulator. Avoiding environmental obstacle(s) is an important issue in the motion planning of redundant robot manipulators. Many effective obstacle avoidance schemes have thus been developed for redundant robot manipulators (Maciekewski and Klein, 1985; Wang and Hamam, 1992; Chen et al., 2002; Lee and Buss, 2007; Guo and Zhang, 2012, 2014; Marcos et al., 2012; Chen and Zhang, 2016; Xiao and Zhang, 2016; Guo and Li, 2016). For example, Lee and Buss (2007) investigated obstacle avoidance by using the Jacobian transpose method. In Marcos et al. (2012), Machado et al. presented a multi-objective method for redundant robot manipulators to avoid obstacles. Note that most reported obstacle avoidance schemes are assumed to be free of noise in the simulations or experiments.

Given its practical application in the industry, another important issue for redundant robot manipulators that requires consideration is the simultaneous handling of environmental noise(s) during the end-effector task execution (Yildirim and Eski, 2010). Noise is inevitably encountered when implementing the scheme for obstacle avoidance of redundant robot manipulators; this noise comes in the form of truncation error, rounding error, model uncertainty, and external disturbance (Jin et al., 2017a; Li et al., 2018). The robustness against additive noise is an important factor in designing a reliable obstacle avoidance scheme. Many researchers have thus examined robot manipulators in the presence of noise (Gaudiano et al., 1996; Florchinger, 2000; Siu et al., 2010; Yildirim and Eski, 2010; Ting et al., 2011; Guo et al., 2017; Jin et al., 2017a; Li et al., 2018). For example, Yildirim and Eski (2010) presented a noise analysis of robot manipulators using neural networks. In Li et al. (2018) designed a new approach for redundant robot manipulators that was combined with the neural controller inherently tolerant to noise.

The aforementioned noise (e.g., realization error and/or external error) has significant effects on the efficacy of the obstacle avoidance schemes for redundant robot manipulators. Such noise may also cause scheme invalidation or the failure of the end-effector primary task. For time-critical end-effector tasks, denoising must be integrated with motion planning for redundant robot manipulators (Li et al., 2018). Time is precious for the obstacle avoidance of redundant robot manipulators in practice, and any noise-reduction preprocessing may consume additional time (which may be in breach of the real-time requirement) (Li et al., 2018). Thus, an effective obstacle avoidance scheme is worth investigating for redundant robot manipulators in a noisy environment. Such a scheme must be inherently tolerant to various types of noise and able to make the robot manipulator avoid obstacles simultaneously.

In recent years, neural dynamics (as an important branch of artificial intelligence) has attracted considerable attention (Zhang et al., 2002; Zhang and Yi, 2011; He et al., 2014a,b; Zhang and Guo, 2015; Li et al., 2016, 2017a,b). It has been studied for solving different mathematical problems arising in motion planning of redundant robot manipulators. In particular, an exponent-type design formula was proposed by Zhang et al. (Zhang et al., 2002; Zhang and Yi, 2011; Zhang and Guo, 2015), and different neural-dynamics models were further developed to solve various types of time-varying problem. Note that some of these models have been applied effectively to redundant robot manipulators, showing well the application prospect of the neural-dynamics approach. By following the aforementioned successful work, another neural-dynamics design formula, which has noise suppression capability, was proposed and investigated by Jin et al. (2016a,b, 2017b). In this paper, such a design formula is exploited to develop an effective obstacle avoidance scheme for motion planning of redundant robot manipulators in the presence of noise.

Specifically, by using the neural-dynamics design formula in Jin et al. (2016a,b, 2017b) to solve the system of time-varying nonlinear kinematic equations, the corresponding obstacle avoidance scheme with the pseudoinverse-type formulation is proposed for redundant robot manipulators in this paper. Such an obstacle avoidance scheme can suppress constant and bounded time-varying noises, and is thus termed as the noise-tolerant obstacle avoidance (NTOA) scheme. For the proposed NTOA scheme, theoretical results are presented to show its excellent property. Computer simulation results are illustrated based on the PA10 robot manipulator with point and window-shaped obstacles to further substantiate the efficacy and superiority of the proposed NTOA scheme for motion planning of redundant robot manipulators.

The rest of this paper is organized into five sections. In section 2, the formulations of the neural-dynamics design formula and the NTOA scheme are given. Section 3 presents the theoretical results of the proposed NTOA scheme. In section 4, simulation results that are synthesized by the proposed NTOA scheme are provided. Section 5 shows the discussion about the NTOA scheme. Section 6 concludes this paper with final remarks. The main contributions of this paper are as follows.

1. This paper proposes and investigates a new NTOA scheme for the motion planning of redundant robot manipulators in the presence of noise. This scheme has a noise-suppressing capability, which can make the robot manipulators achieve their obstacle-avoidance purpose. This paper marks an important advancement in obstacle avoidance research by proposing and providing a NTOA scheme.
2. In this paper, the Cartesian error synthesized by the proposed NTOA scheme is proven to possess the property of global and exponential convergence. Theoretical results also indicate that the proposed scheme has the capability of suppressing constant and bounded time-varying noises.
3. Computer simulations based on the PA10 robot manipulator are performed to substantiate the efficacy and superiority of the proposed NTOA scheme whether in the presence or absence of noise.

## 2. Design formula and scheme formulation

In this section, the formulation of the neural-dynamics design formula is presented. Then, by exploiting this design formula to solve the system of time-varying nonlinear kinematic equations, the corresponding obstacle avoidance scheme is developed for motion planning of redundant robot manipulators in the presence of noise.

### 2.1. Neural-dynamics design formula

Let us consider the following time-varying problem:

$$\begin{array}{l} \phi \left(t\right)=0\in R^{n}. \end{array}$$

In the neural-dynamics design methodology (Zhang et al., 2002; Zhang and Yi, 2011; Zhang and Guo, 2015), to solve this problem, the following vector-valued error function is defined:

$$\begin{array}{l} e\left(t\right):=\phi \left(t\right)\in R^{n}. \end{array}$$

Then, the time derivative of the error function *e*(*t*), i.e., ė(*t*), is selected such that *e*(*t*) converges to zero. In Zhang et al. (2002), an exponent-type design formula was originally developed by Zhang et al. to determine ė(*t*). Such a design formula is written as follows:

(1) $$\begin{array}{r} ė\left(t\right)=-\gamma e\left(t\right), \end{array}$$

where design parameter γ>0∈*R* is used to scale the convergence rate of the solution (Zhang et al., 2002; Zhang and Yi, 2011; Zhang and Guo, 2015). Corresponding to the specific time-varying problem to be solved, expanding (1) yields the related neural-dynamics model. Furthermore, based on (1), another neural-dynamics design formula with noise suppression capability was developed by Jin et al. (2016a,b, 2017b). This design formula is written as follows:

(2) $$\begin{array}{r} ė\left(t\right)=-k_{\text{P}}e\left(t\right)-k_{\text{I}}\int_{0}^{t}e\left(\tau \right)\text{d}\tau , \end{array}$$

where *k*_P_>0∈*R* and *k*_I_>0∈*R* are the design parameters. Please refer to Jin et al. (2016a,b, 2017b) for details about the property of (2). Based on previous research, such a neural dynamics design formula (2) is applied to the obstacle avoidance of redundant robot manipulators in the ensuing section.

### 2.2. NTOA scheme

The redundancy-resolution problem related to the motion planning of redundant robot manipulators is described as follows: given the desired end-effector path $r_{\text{d}}\left(t\right)\in R^{m}$, the corresponding joint trajectory θ(*t*)∈*R*^*n*^ should be obtained in real time *t*. In mathematics, solving the redundancy-resolution problem can be equivalent to solving the following system of time-varying nonlinear kinematic equations:

(3) $$\begin{array}{r} f\left(\theta \right)=r_{\text{d}}, \end{array}$$

where *f*(·) is a differentiable nonlinear mapping.

To solve (3), the error function *e*(*t*) is defined as follows:

$$\begin{array}{l} e\left(t\right)=f\left(\theta \right)-r_{\text{d}}\in R^{m}. \end{array}$$

Evidently, expanding (2) yields the following result:

(4) $$\begin{array}{r} J\overset{{}^{\circ}}{\theta }={\dot{r}}_{\text{d}}-k_{\text{P}}\left(f\left(\theta \right)-r_{\text{d}}\right)-k_{\text{I}}\int_{0}^{t}\left(f\left(\theta \right)-r_{\text{d}}\right)\text{d}\tau , \end{array}$$

where *J*∈*R*^*m*×*n*^ is the Jacobian matrix of the robot manipulator, $\overset{{}^{\circ}}{\theta }\in R^{n}$ is the joint-velocity vector, and ${ṙ}_{\text{d}}\in R^{m}$ is the time derivative of *r*_d_. By generalizing the conventional pseudoinverse-type formulation (Siciliano and Khatib, 2008; Siciliano et al., 2009), the following redundancy-resolution scheme for the motion planning of redundant robot manipulators is obtained:

(5) $$\begin{array}{r} \overset{{}^{\circ}}{\theta }=J^{†}\left({\dot{r}}_{\text{d}}-k_{\text{P}}\left(f\left(\theta \right)-r_{\text{d}}\right)-k_{\text{I}}\int_{0}^{t}\left(f\left(\theta \right)-r_{\text{d}}\right)\text{d}\tau \right)+\left(I-J^{†}J\right)z. \end{array}$$

As mentioned previously, avoiding environmental obstacle(s) is an important issue in the motion planning of redundant robot manipulators. Thus, by choosing a suitable *z* in (5), the NTOA scheme proposed in this paper for redundant robot manipulators is formulated as follows:

(6) $$\begin{array}{r} \overset{{}^{\circ}}{\theta }=J^{†}\left({\dot{r}}_{\text{d}}-k_{\text{P}}\left(f\left(\theta \right)-r_{\text{d}}\right)-k_{\text{I}}\int_{0}^{t}\left(f\left(\theta \right)-r_{\text{d}}\right)\text{d}\tau \right)+\kappa \overset{n-\varsigma }{\underset{i=1}{\sum }}V_{Ni}^{\text{T}}\nabla H\left(\theta \right)V_{Ni}, \end{array}$$

where κ∈*R* is a real scalar (Li et al., 2001), ς = rank(*J*), and superscript ^T^ denotes the transpose operator. In addition, ∇*H*(θ) is the gradient of a performance criterion *H*(θ) (being a scalar function of the joint-angle vector θ∈*R*^*n*^), and *V*_*Ni*_ is the *i*th column vector of $V_{N}\in R^{n\times \left(n-\varsigma \right)}$ with *V*_*N*_ = [*V*_ς+1_*V*_ς+2_ ⋯*V*_*n*_]. *V*_*j*_ (with *j* = ς+1, ς+2, ⋯ , *n*) is the *j*th column vector of the orthogonal unitary matrix *V*∈*R*^*n*×*n*^, which is obtained by using the singular value decomposition (SVD) of *J* (Lee and Buss, 2007). That is,

$$\begin{array}{l} J=USV^{\text{T}}, \end{array}$$

where *U*∈*R*^*m*×*m*^ is the orthogonal matrix and *S*∈*R*^*m*×*n*^ contains the singular values of *J* in its main diagonal. For obstacle avoidance, ∇*H*(θ) in (6) is replaced with the following escape velocity ${\overset{{}^{\circ}}{\theta }}_{C}$ in the joint space (Lee and Buss, 2007):

$$\begin{array}{l} {\overset{{}^{\circ}}{\theta }}_{C}=\overset{k}{\underset{i=1}{\sum }}J_{Ci}^{\text{T}}\left(\theta \right)v_{Cij}, \end{array}$$

where *k* is the number of critical points and *J*_*Ci*_(θ) is the Jacobian matrix of the *i*th critical point *C*_*i*_. In addition, *v*_*Cij*_ is defined as a function of the minimum distance *d*_*ij*_ (between the *i*th link and the *j*th obstacle) along the direction away from the critical point *C*_*i*_ (Lee and Buss, 2007):

$$v_{Cij}=\{\begin{array}{ll} 0 & \text{for}\;d_{1}<d_{ij},\\ \frac{v_{0}}{2}\{\cos(\pi \frac{d_{ij}-d_{2}}{d_{1}-d_{2}})+1\}u_{ij} & \text{for}\;d_{2}<d_{ij}⩽d_{1},\\ v_{0}u_{ij} & \text{for}\;d_{ij}⩽d_{2}, \end{array}$$

where *v*_0_ is the maximum escape velocity and *u*_*ij*_ is the unit vector from the critical point *C*_*i*_ on the *i*th link to the *j*th obstacle. In addition, the predefined thresholds *d*_1_ and *d*_2_ are the outer and inner safety thresholds, respectively. Descriptions of the outer and inner safety thresholds can be seen in Guo and Zhang (2014) and/or Guo and Li (2016).

Note that, for the proposed NTOA scheme (6), this paper limits the investigation that the two design parameters *k*_P_ and *k*_I_ satisfy $k_{\text{P}}^{2}>4k_{\text{I}}$ numerically. Furthermore, theoretical results of (6) are presented in the ensuing section to show its effectiveness on motion planning and noise suppression.

## 3. Theoretical results

In this section, four theorems are provided to investigate the performance of the proposed NTOA scheme (6) in three situations, namely, the zero noise, constant noise, and bounded time-varying noise.

### 3.1. Obstacle avoidance without considering noise

In this subsection, the proposed NTOA scheme (6) is studied for redundant robot manipulators without considering the existence of noise (i.e., zero noise situation).

**Theorem 1:** *The trajectory of the Cartesian error e*(*t*) *for the proposed NTOA scheme (6) is asymptotically stable*.

**Proof:** See Appendix A in Supplementary Material.

**Theorem 2:** *In addition to Theorem 1, the Cartesian error e*(*t*) *synthesized by the proposed NTOA scheme (6) has the property of exponential convergence*.

**Proof:** See Appendix B in Supplementary Material.

For the proposed NTOA scheme (6), the results of Theorems 1 and 2 indicate that the corresponding Cartesian error *e*(*t*) has the property of global and exponential convergence. This means that the magnitude of *e*(*t*) synthesized by (6) can be kept within the region of a small value. By choosing the *k*_P_ and *k*_I_ values appropriately, the *e*(*t*) magnitude can be small enough during the motion task execution (whether noise exists or not), then the performance of (6) is considered satisfactory (De Luca et al., 1992). In summary, these results theoretically guarantee that the proposed NTOA scheme (6) is effective in the motion planning of redundant robot manipulators.

### 3.2. Obstacle avoidance with noise considered

In this subsection, the proposed NTOA scheme (6) is investigated considering the existence of noise (i.e., the situations of constant noise and bounded time-varying noise). For further investigation, the proposed NTOA scheme (6) under the pollution of noise is formulated as follows:

(7) $$\begin{array}{c} \overset{{}^{\circ}}{\theta }=J^{†}({\dot{r}}_{\text{d}}-k_{\text{P}}(f(\theta )-r_{\text{d}})-k_{\text{I}}{\int^{\text{​}}}_{0}^{t}(f(\theta )-r_{\text{d}})\text{d}\tau +\delta (t)+\kappa \overset{i=1}{\underset{n-\varsigma }{\sum^{\text{​}}}}V_{Ni}^{T}\nabla H(\theta )V_{Ni}, \end{array}$$

where δ(*t*)∈*R*^*m*^ denotes the vector-form noise (e.g., constant realization errors, time-varying bias errors, and the superposition of these errors). Now, the performance of the noise-polluted NTOA scheme (7) is studied for redundant robot manipulators.

**Theorem 3:** *In the case of the vector-form constant noise* δ(*t*) = *c*∈*R*^*m*^*, the Cartesian error e*(*t*) *synthesized by the noise-polluted NTOA scheme (7) has a convergence property*.

**Proof:** See Appendix C in Supplementary Material.

As mentioned before, the convergence property can guarantee a small magnitude of the Cartesian error *e*(*t*) during the motion task execution. Based on the proof in Appendix C in Supplementary Material, no matter how large the unknown vector-form constant noise δ(*t*) = *c*∈*R*^*m*^ is, the Cartesian error *e*(*t*) for the noise-polluted NTOA scheme (7) is convergent, with the steady-state error being zero. Thus, as synthesized by (7) with the appropriate values of *k*_P_ and *k*_I_, the magnitude of *e*(*t*) can be small enough.

In many practical applications, the noise can be time-varying. For the fast time-varying noise, the low-pass filter could be exploited to tackle the problem of error compensation. However, the noise-reduction preprocessing may consume extra time and violate the real-time requirement. Thus, investigating the performance of the noise-polluted NTOA scheme (7) in the presence of bounded time-varying noise (or even random noise) is necessary.

**Theorem 4:** *In the case of the bounded vector-form time-varying noise* δ(*t*)∈*R*^*m*^*, the Cartesian error e*(*t*) *synthesized by the noise-polluted NTOA scheme (7) is bounded, with the steady-state error being bounded by*

$$\begin{array}{l} 2\sqrt{\frac{m}{k_{\text{P}}^{2}-4k_{\text{I}}}}\underset{0\leq \tau \leq t}{\max}|\delta_{i}\left(\tau \right)|, \end{array}$$

*where* δ_*i*_(*t*) *denotes the ith element of* δ(*t*) *and the symbol* |·| *denotes the absolute value of a scalar*.

**Proof:** See Appendix D in Supplementary Material.

From Theorem 4, the upper bound of the steady-state error is in inverse proportion to the *k*_P_ value. By choosing a large enough *k*_P_ value and an appropriate *k*_I_ value, the steady-state error can be arbitrarily small, which means that the magnitude of *e*(*t*) synthesized by (7) can also be small enough during the task execution. This result further shows that the noise-polluted NTOA scheme (7) is effective in the motion planning of redundant robot manipulators in the presence of bounded time-varying noise.

In summary, the results in this section (i.e., Theorems 1–4) theoretically substantiate the efficacy and superiority of the proposed NTOA scheme (6) for redundant robot manipulators whether noise exists or not.

## 4. Simulative verifications

In this section, based on the PA10 robot manipulator with an equipped long tool (Zhang and Wang, 2004; Guo and Zhang, 2012, 2014), computer simulations with the existence of point and window-shaped obstacles are performed to verify the efficacy and superiority of the proposed NTOA scheme (6). For comparison, the obstacle avoidance method presented in Lee and Buss (2007) is simulated as well.

### 4.1. Point obstacle avoidance

In this example, the PA10 robot manipulator is simulated, in which there exists a point obstacle. The obstacle avoidance method in Lee and Buss (2007) and the proposed NTOA scheme (6) are both applied to such a robot manipulator for the end-effector tracking of a circular path considering three situations (i.e., the situations of zero, constant, and bounded time-varying noises).

#### 4.1.1. Zero noise

**Figure 1:**
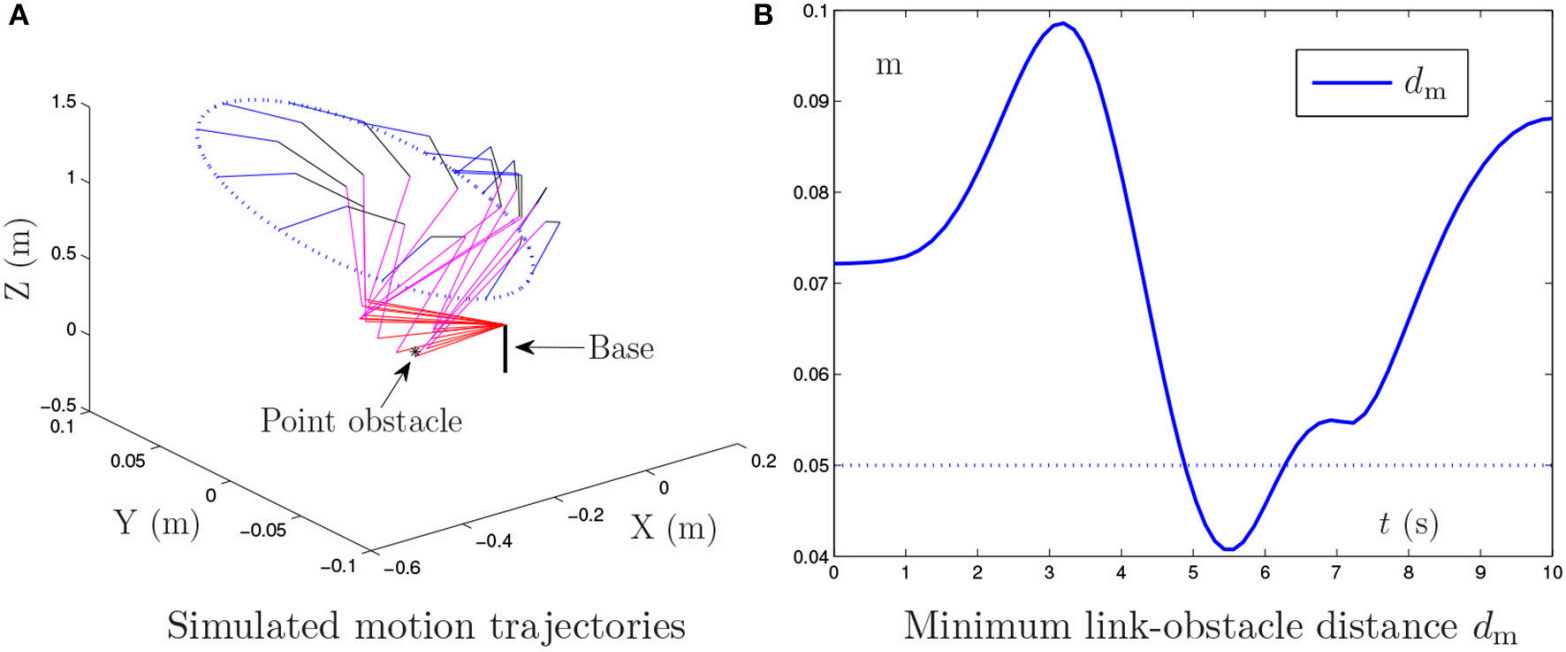
Simulation results synthesized by the general MVN scheme [i.e., the proposed NTOA scheme (6) using *k*_P_ = *k*_I_ = κ = 0] for the PA10 end-effector tracking the circular path, where the existence of the point obstacle and noise is not considered.

**Figure 2:**
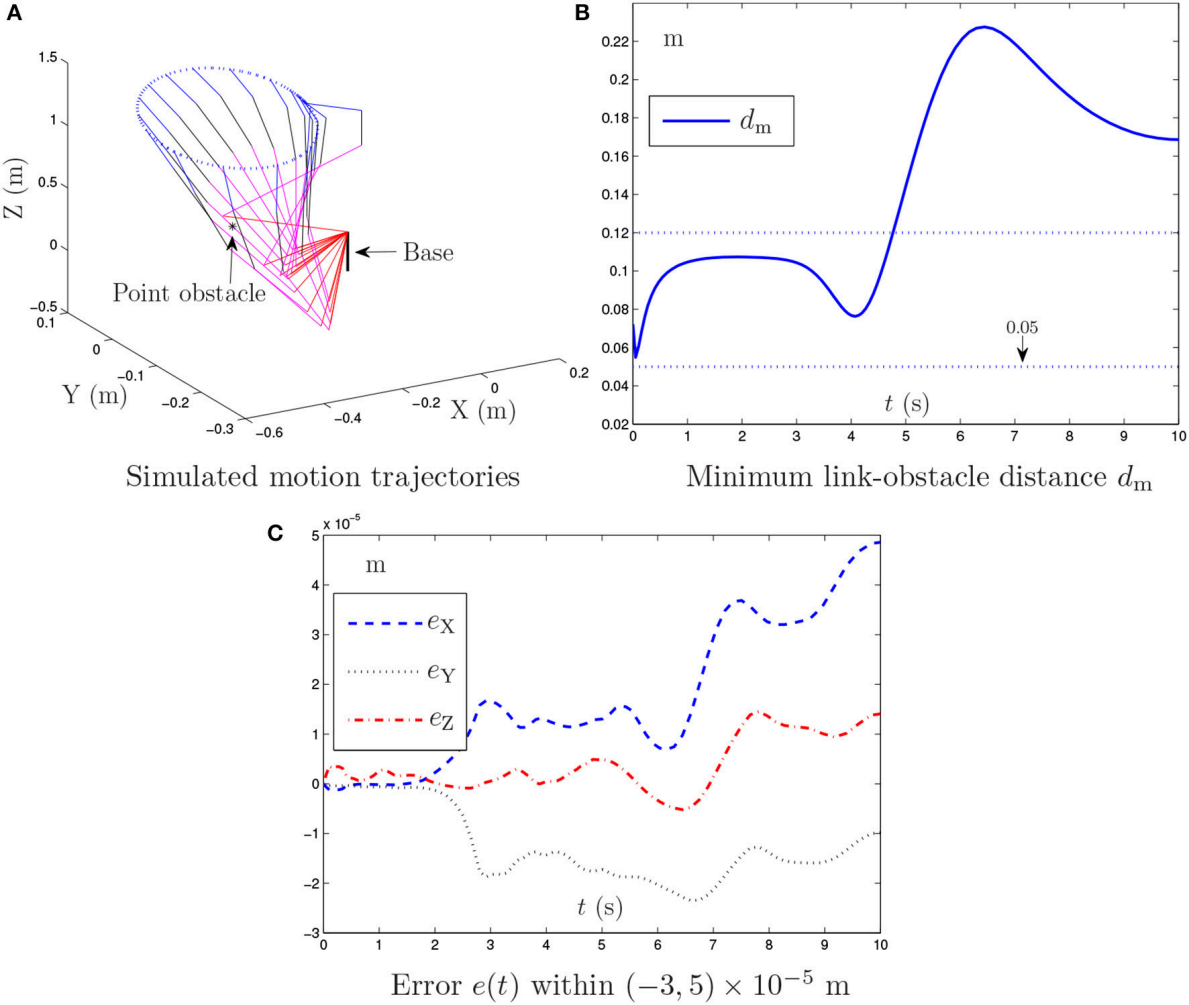
Simulation results synthesized by the obstacle avoidance method in Lee and Buss (2007) for the PA10 end-effector tracking the circular path, where the zero noise is considered.

**Figure 3:**
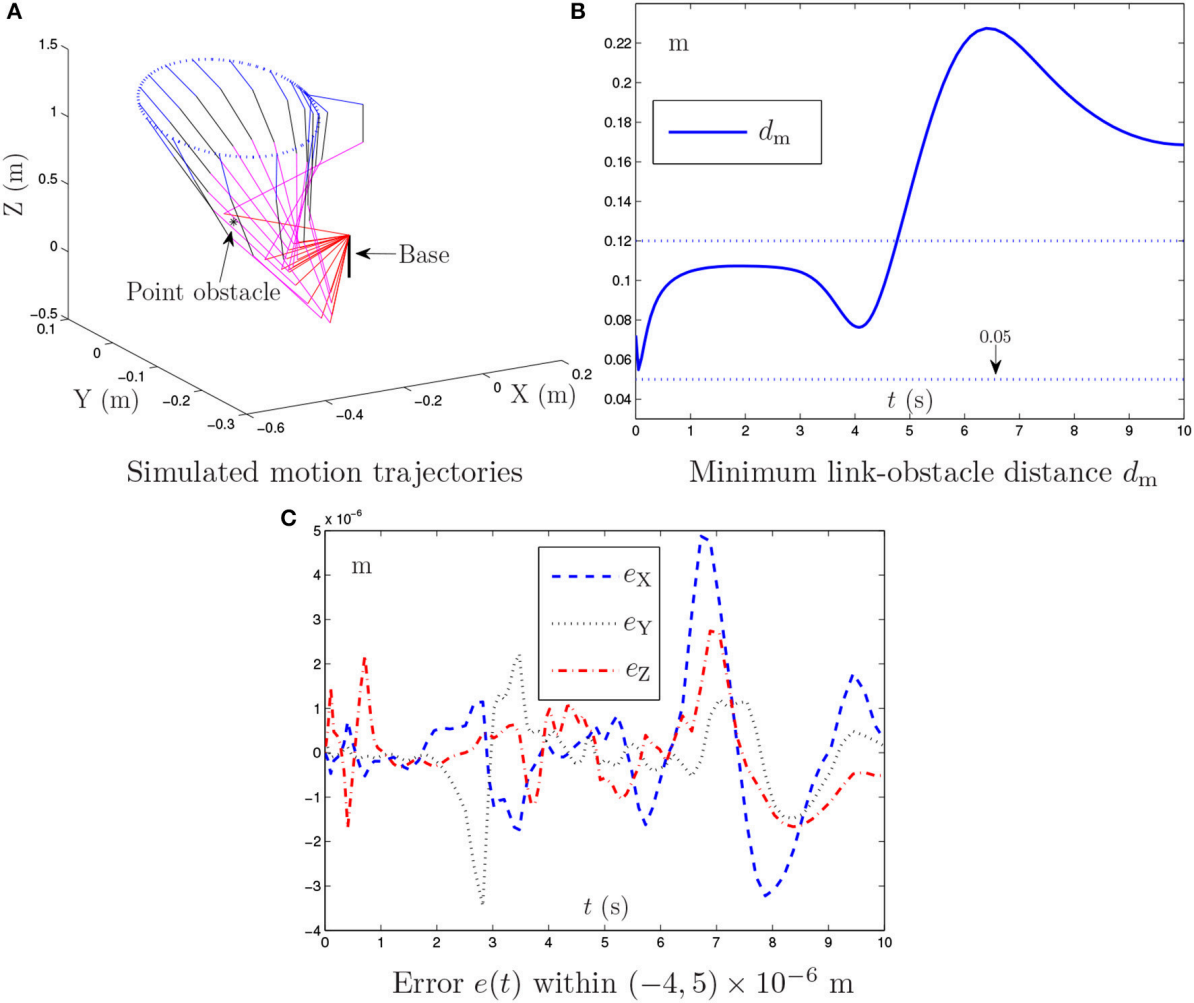
Simulation results synthesized by the proposed NTOA scheme (6) using *k*_P_ = *k*_I_ = 10 and κ = 1 for the PA10 end-effector tracking the circular path, where the zero noise is considered.

The obstacle avoidance method in Lee and Buss (2007) and the proposed NTOA scheme (6) are both tested in the zero-noise situation, and the corresponding simulation results are presented in Figures 1–3 and Table 1.

**Table 1:** Maximal absolute value (MAV) of Cartesian error synthesized by the proposed NTOA scheme (6) with zero noise considered and with different *k*_P_ and *k*_I_ values used.

**#**	**MAV of *e*_X_**	**MAV of *e*_Y_**	**MAV of *e*_Z_**
*k*_P_ = *k*_I_ = 10	4.874 × 10^−6^	3.449 × 10^−6^	2.741 × 10^−6^
*k*_P_ = *k*_I_ = 100	4.138 × 10^−7^	1.052 × 10^−6^	9.202 × 10^−7^
*k*_P_ = *k*_I_ = 1000	2.392 × 10^−7^	3.708 × 10^−7^	4.321 × 10^−7^

Figure 1 shows the simulation results synthesized by the general minimum velocity norm (MVN) scheme [i.e., the proposed NTOA scheme (6) using *k*_P_ = *k*_I_ = κ = 0], in which the existence of the point obstacle is not considered. As seen from Figure 1A, the desired motion is achieved successfully by the PA10 robot manipulator. However, Figure 1B indicates that the minimum link-obstacle distance *d*_m_ is smaller than 0.05 m during [4.91, 6.25] s. This can be viewed as a collision phenomenon (Guo and Zhang, 2014; Guo and Li, 2016) that may cause damage to the robot manipulator and the point obstacle. Thus, exploiting an effective obstacle avoidance method/scheme for PA10 robot manipulator is necessary.

Figure 2 illustrates the simulation results synthesized by the obstacle avoidance method in Lee and Buss (2007), where the existence of the point obstacle is considered. As shown in Figures 2A,B, during the motion task execution, the minimum link-obstacle distance *d*_m_ is always greater than 0.05 m. This substantiates that the presented point obstacle is successfully avoided by the obstacle avoidance method in Lee and Buss (2007). Figure 2C shows that the maximal Cartesian error is approximately 5.0 × 10^−5^ m. However, the detailed results in Figure 2C show that the divergence phenomenon in the Cartesian error is present. Thus, the obstacle avoidance method in Lee and Buss (2007), although effective, may be less desirable and less applicable in robotics.

By contrast, Figure 3 presents the simulation results synthesized by the proposed NTOA scheme (6) with *k*_P_ = *k*_I_ = 10 and κ = 1. As seen from Figures 3A,B, the minimum link-obstacle distance *d*_m_ during the motion is always >0.05 m, which implies that obstacle avoidance is achieved successfully. In addition, as shown in Figure 3C, the maximal Cartesian error is < 5.0 × 10^−6^ m, indicating the efficacy of the proposed NTOA scheme (6) for the motion planning of PA10 robot manipulator. The comparison between Figure 2C and Figure 3C shows that the maximal Cartesian error via (6) is about 10 times smaller than the one via the obstacle avoidance method in Lee and Buss (2007). Furthermore, the divergence phenomenon does not exist for the Cartesian error presented in Figure 3C. Thus, a prominent advantage of the proposed NTOA scheme (6) is that it guarantees a Cartesian error with no divergence (which agrees with the convergence results in Theorems 1 and 2). These comparative results indicate that the proposed NTOA scheme (6) is superior to the obstacle avoidance method in Lee and Buss (2007).

For further investigation, the proposed NTOA scheme (6) is tested by using different *k*_P_ and *k*_I_ values, and the related simulation results are presented in Table 1. As seen from Table 1, the maximal absolute value (MAV) of the Cartesian error synthesized by (6) is small enough (i.e., of order 10^−7^~10^−6^), showing the efficacy on motion planning. In addition, it follows from Table 1 that the MAV of Cartesian error decreases when the *k*_P_ and *k*_I_ values increase. Thus, the design parameters *k*_P_ and *k*_I_ play an important role in (6), and should be set as large as the robotics systems would permit (or selected appropriately large for simulation/experiment purposes).

In summary, the above results (i.e., Figures 1–3 and Table 1) substantiate the efficacy and superiority of the proposed NTOA scheme (6), as compared with the obstacle avoidance method in Lee and Buss (2007).

#### 4.1.2. Constant noise

**Figure 4:**
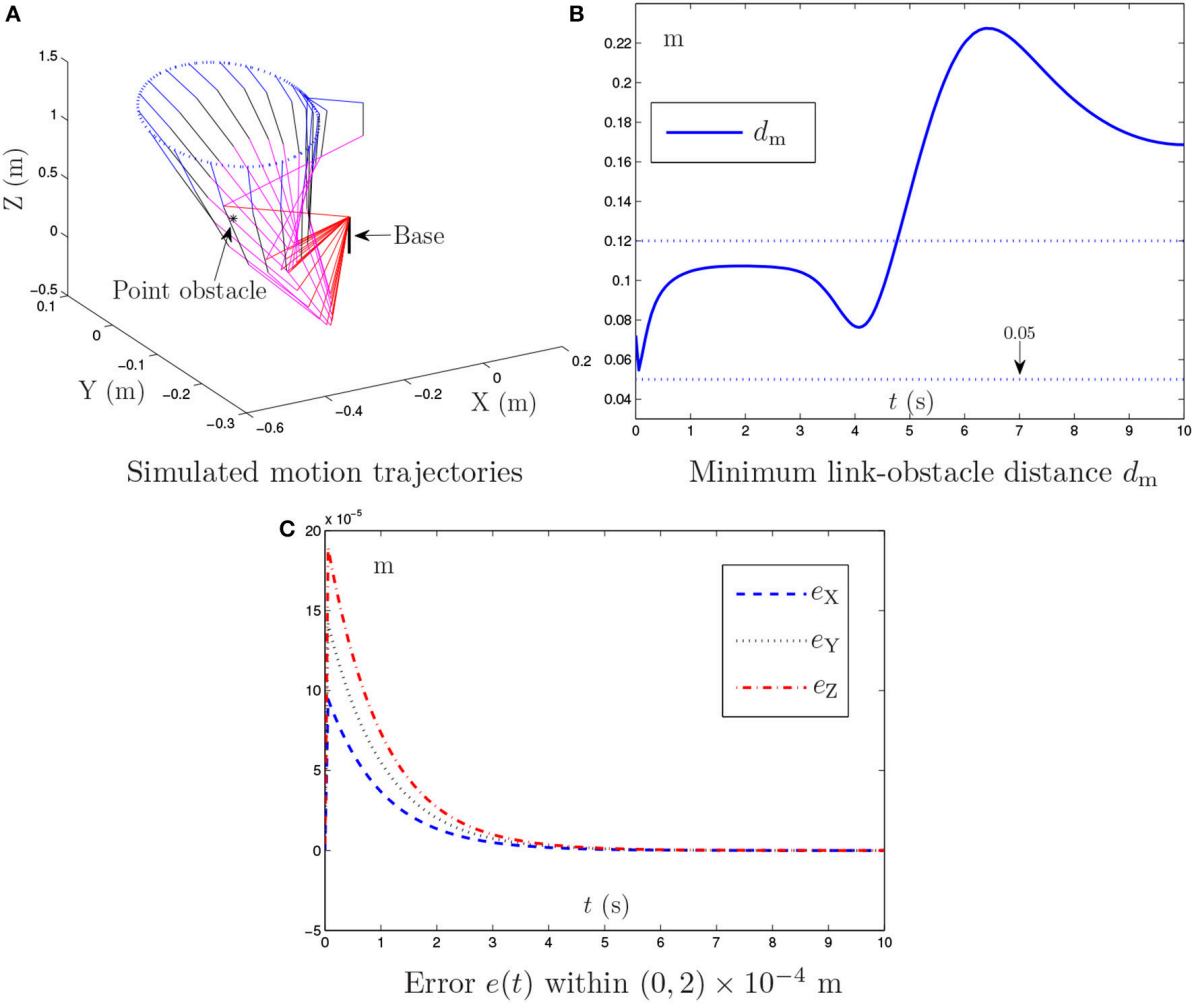
Simulation results synthesized by the proposed NTOA scheme (6) using $k_{\text{P}}=k_{\text{I}}=10^{3}$ and κ = 1 for the PA10 end-effector tracking the circular path, where the constant noise is considered.

The proposed NTOA scheme (6) is tested in the situation of the constant noise δ(*t*) = *c* = [0.10, 0.15, 0.20]^T^. In this situation, the noise-polluted NTOA scheme (7) is actually used, and the corresponding simulation results are shown in Figure 4 and Table 2. Note that the obstacle avoidance method in Lee and Buss (2007) with constant noise considered is test as well. However, the computer simulation failed, which shows that such a method does not have a capability of suppressing noise and cannot handle this kind of noise (thereby leading to the failure of the task execution).

**Table 2:** Maximal absolute value (MAV) of Cartesian error synthesized by the proposed NTOA scheme (6) with constant noise considered and with different *k*_P_ and *k*_I_ values used.

**#**	**MAV of *e*_X_**	**MAV of *e*_Y_**	**MAV of *e*_Z_**
$k_{\text{P}}=k_{\text{I}}=10^{3}$	9.457 × 10^−5^	1.419 × 10^−4^	1.893 × 10^−4^
$k_{\text{P}}=k_{\text{I}}=10^{4}$	9.466 × 10^−6^	1.421 × 10^−5^	1.893 × 10^−5^
$k_{\text{P}}=k_{\text{I}}=10^{5}$	9.441 × 10^−7^	1.425 × 10^−6^	1.907 × 10^−6^

Figure 4 shows the simulation results synthesized by the noise-polluted NTOA scheme (7) with $k_{\text{P}}=k_{\text{I}}=10^{3}$ and κ = 1. As seen from Figure 4, the obstacle-avoidance purpose is achieved successfully via (7) in the sense that *d*_m_ is always >0.05 m. In addition, the simulated end-effector trajectory is close to the desired circular path with a small Cartesian error. The existence of constant noise leads to the significant increase of the Cartesian error (from the zero initial value) in the transient phase, as shown in Figure 4C. Owing to the noise suppressing capability, (7) can handle this kind of noise. The resultant Cartesian error is convergent (which agrees with the result of Theorem 3), and is kept within the region of a small value (i.e., of order 10^−4^). These results indicate that the noise-polluted NTOA scheme (7) is effective for robotic practical applications.

The noise-polluted NTOA scheme (7) is tested as well using different *k*_P_ and *k*_I_ values, and the related simulation results are presented in Table 2. As shown in Table 2, the MAV of Cartesian error is small enough, meaning that the motion planning task is executed successfully via (7) (though constant noise exists). Table 2 also indicates that the performance of (7) is improved effectively by increasing the *k*_P_ and *k*_I_ values, showing again the important role of *k*_P_ and *k*_I_ in the noise-polluted NTOA scheme (7).

In summary, the above simulation results substantiate the efficacy and superiority of the proposed NTOA scheme (6) [i.e., the performance of the noise-polluted NTOA scheme (7)] for motion planning of redundant robot manipulators in the presence of constant noise.

#### 4.1.3. Bounded time-varying noise

**Figure 5:**
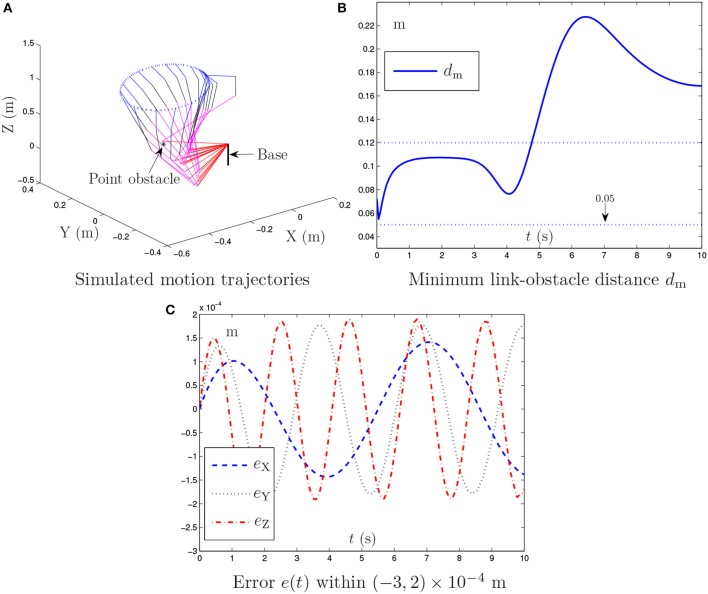
Simulation results synthesized by the proposed NTOA scheme (6) using $k_{\text{P}}=k_{\text{I}}=10^{3}$ and κ = 1 for the PA10 end-effector tracking the circular path, where the bounded time-varying noise is considered.

The proposed NTOA scheme (6) is tested in the situation of the bounded time-varying noise δ(*t*) = [0.2sin(*t*), 0.2sin(2*t*), 0.2sin(3*t*)]^T^. Similarly, the noise-polluted NTOA scheme (7) is actually used in this situation, and the corresponding simulation results are presented in Figure 5.

As shown in Figure 5, the minimum link-obstacle distance *d*_m_ is always >0.05 m and the maximal Cartesian error is < 3.0 × 10^−4^ m. Thus, the purposes of obstacle avoidance and motion planning are both achieved successfully using the noise-polluted NTOA scheme (7). According to Figure 5C, the Cartesian error is bounded and is kept within (−3, 2) × 10^−4^ m during the task execution. This coincides with the result of Theorem 4. The noise-polluted NTOA scheme (7) is tested using different *k*_P_ and *k*_I_ values for further investigation. Owing to the similarity of results, the related simulation results are omitted here. This result indicates that the MAV of the Cartesian error via (7) decreases as the *k*_P_ and *k*_I_ values increase.

In summary, these simulation results substantiate the efficacy and superiority of the proposed NTOA scheme (6) [or the excellent performance of the noise-polluted NTOA scheme (7)] for redundant robot manipulators in the presence of bounded time-varying noise.

### 4.2. Window-shaped obstacle avoidance

In this example, the PA10 robot manipulator is simulated, in which there exists a window-shaped obstacle (Guo and Zhang, 2014). The proposed NTOA scheme (6) is applied to such a robot manipulator for its end-effector tracking a circular path. Similarly, the following three situations of noise are considered in the investigation of the proposed NTOA scheme (6):

$$\{\begin{array}{l} \text{Zero noise:}\;{\left[0,0,0\right]}^{\text{T}},\\ \text{Constant noise:}\;{\left[0.1,0.2,0.3\right]}^{\text{T}},\\ \text{Bounded time-varying noise:}\;{\left[0.2\cos(t),0.2\cos(2t),0.2\cos(3t)\right]}^{\text{T}}. \end{array}$$

**Figure 6:**
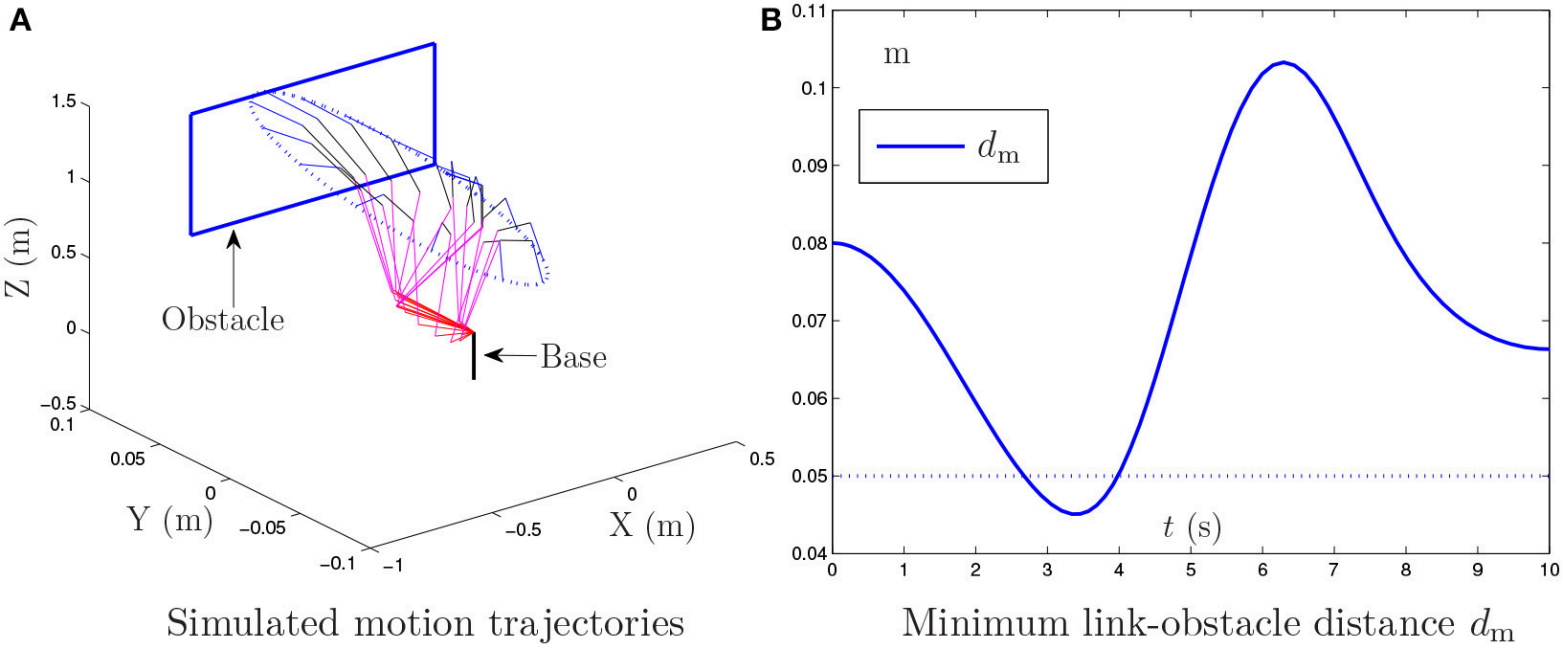
Simulation results synthesized by the general MVN scheme for the PA10 end-effector tracking the circular path, where the existence of the window-shaped obstacle and noise is not considered.

Without considering the existence of the window-shaped obstacle, Figure 6 shows the simulation results synthesized by the general MVN scheme. As seen from Figure 6, the desired motion for the PA10 robot manipulator is achieved successfully, but the minimum link-obstacle distance *d*_m_ is smaller than 0.05 m during [2.67, 3.96] s. This close distance (being < 0.05 m) means that there exists a collision that may cause considerable damage to the PA10 robot manipulator and the window-shaped obstacle.

**Figure 7:**
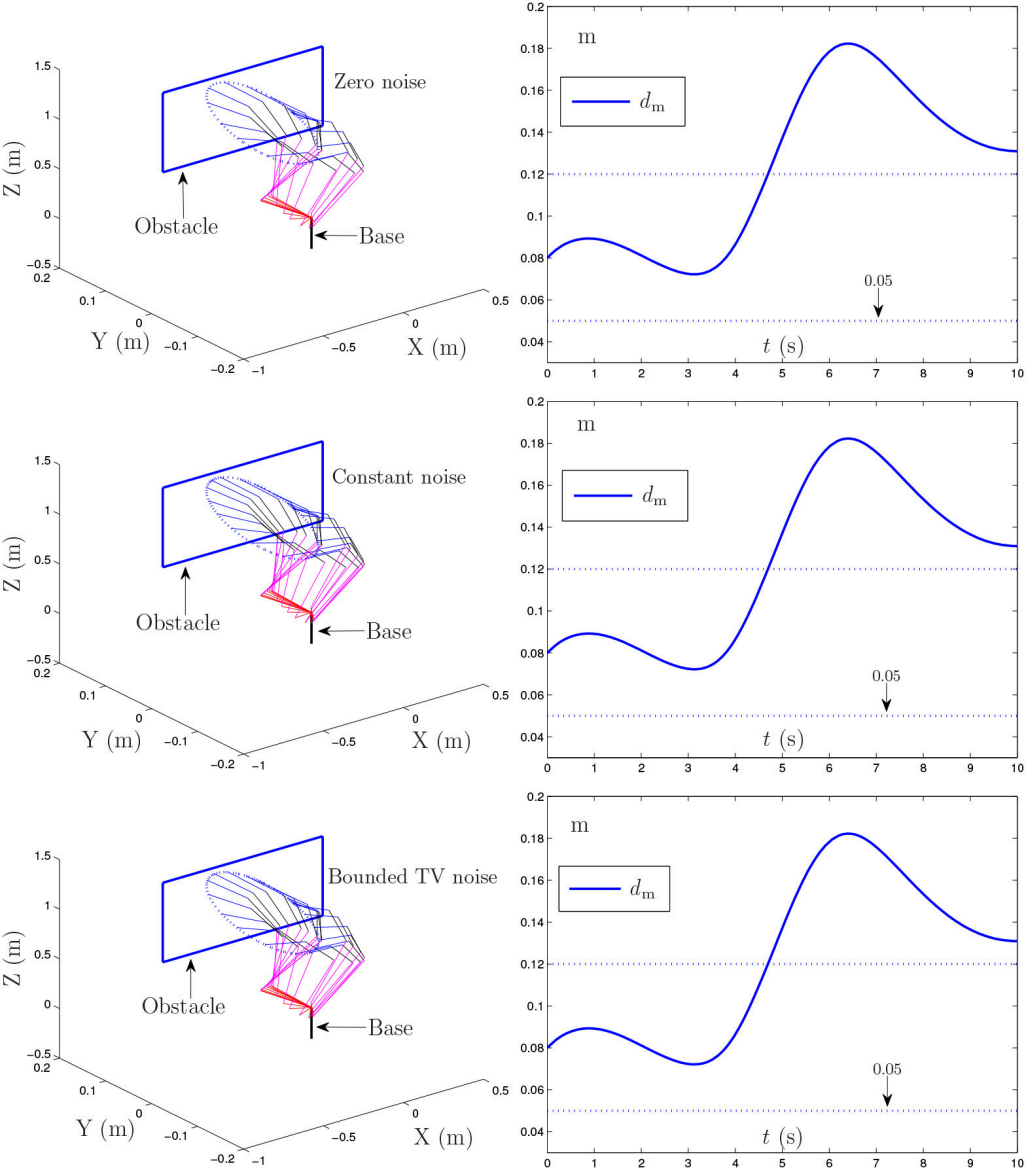
Simulation results synthesized by the proposed NTOA scheme (6) for the PA10 end-effector tracking the circular path, where the zero noise, constant noise, and bounded time-varying (TV) noise are considered.

**Figure 8:**
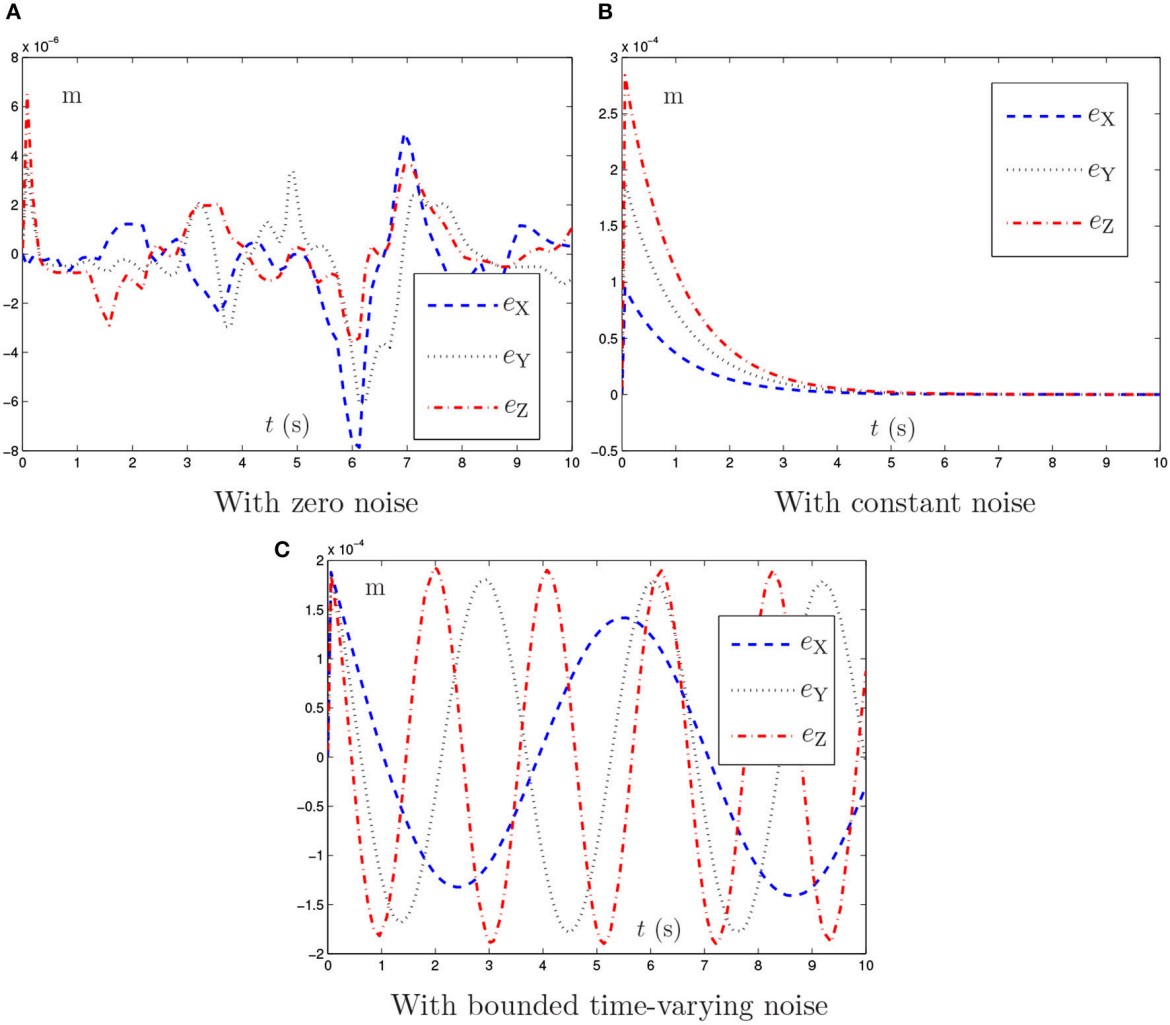
Trajectories of Cartesian error *e*(*t*) synthesized by the proposed NTOA scheme (6) with three situations considered, which corresponds to Figure 7.

To avoid the window-shaped obstacle, the proposed NTOA scheme (6) [as well as the noise-polluted NTOA scheme (7)] is applied to the PA10 robot manipulator under the aforementioned three situations, and the simulation results are shown in Figures 7, 8. Note that, for Figure 7, the left subfigures present the simulated motion trajectories of the robot manipulator, and the right subfigures present the corresponding profiles of the minimum link-obstacle distance *d*_m_. Evidently, as shown in Figures 7, 8, all of the simulated end-effector trajectories are close to the desired circular path (with the Cartesian errors being small enough), and the minimum link-obstacle distance *d*_m_ during the motion is always greater than 0.05 m. This indicates that the obstacle-avoidance and motion-planning purposes are both achieved successfully via (6) [or (7)], no matter whether noise exists or not. Besides, as seen from Figure 8, in each situation of noise, the change of the Cartesian error is similar to the change presented in Figures 3C, 4C, 5C, showing that these results coincide with the results of Theorems 1–4. Thus, being one of its prominent advantages, the proposed NTOA scheme (6) guarantees that the Cartesian error occurs without divergence, as evidenced by both the theoretical and simulation results.

In summary, these simulation results have substantiated again the efficacy and superiority of the proposed NTOA scheme (6) for motion planning of redundant robot manipulators in the presence of different kinds of noise.

## 5. Discussion

For the proposed NTOA scheme (6), the feedback item *k*_P_(*f*(θ)−*r*_d_) and the integration item $k_{\text{I}}{\int^{\text{​}}}_{t}^{0}(f(\theta )-r_{\text{d}})\text{d}\tau )$ are incorporated into the scheme formulation. Such a scheme thus contains the proportional, integral, and derivative information of the desired end-effector path *r*_d_. In this sense, the proposed NTOA scheme (6) processes the characteristic of proportional-integral-derivative (PID), thereby showing that (6) can be considered as a nonlinear PID controller for the obstacle avoidance of redundant robot manipulators. Because of the special characteristic, the proposed scheme (6) is robust against constant noise and bounded time-varying noise, and enables the effective obstacle avoidance of redundant robot manipulators even in the presence of noise. The efficacy of (6) has been analyzed and verified via the theoretical and simulation results in sections 3 and 4.

By summarizing the simulation results in section 4, the superiority of the proposed NTOA scheme (6) over the obstacle avoidance method in Lee and Buss (2007) is presented as follows.

- In the situation of zero noise, the obstacle avoidance method in Lee and Buss (2007) would introduce undesirable divergence phenomenon in Cartesian error (see Figure 2). By contrast, the error *e(t)* via the proposed NTOA scheme (6) will not encounter the divergence problem owing to the feedback and integration items in (6). In addition, the motion precision of the proposed NTOA scheme (6) is better than that of the obstacle avoidance method in Lee and Buss (2007), as demonstrated in section 4.
- In the situation of nonzero noise, the obstacle avoidance method in Lee and Buss (2007) is intolerant to noise, which invalidates the method. By contrast, the proposed NTOA scheme (6) can suppress constant noise and bounded time-varying noise (see sections 3 and 4). Thus, the efficacy of the proposed NTOA scheme (6) is theoretically guaranteed even in the presence of noise.

In summary, the proposed NTOA scheme (6) is advantageous over the obstacle avoidance method in Lee and Buss (2007) because it guarantees nondivergence Cartesian error regardless of the absence or presence of noise. Given this characteristic, the proposed NTOA scheme (6) is superior to the obstacle avoidance method in Lee and Buss (2007) for redundant robot manipulators.

Besides, both the theoretical and simulation results in sections 3 and 4 have indicated that the design parameters *k*_P_ and *k*_I_ are important to ensure the precision of the Cartesian error for the proposed NTOA scheme (6). To a certain extent, such two parameters are similar to the PID parameters and can be used to enhance the noise suppression capability of the proposed NTOA scheme (6). In general, the values of *k*_P_ and *k*_I_ can be selected in accordance with the actual situations of noise and practical requirements of precision. Summarizing the theoretical analysis and simulation results shows that *k*_P_ and *k*_I_ should be set to sufficiently large values (e.g., $k_{\text{P}}=k_{\text{I}}=10^{3}$ or larger) to ensure the satisfactory performance of the proposed NTOA scheme (6), particularly in the presence of noise.

## 6. Conclusion

In this paper, based on the neural dynamics design formula in Jin et al. (2016a,b, 2017b), the new NTOA scheme (6) is proposed and investigated for the motion planning of redundant robot manipulators in the presence of noise. This scheme, which is capable of suppressing constant and bounded time-varying noises, enables the robot manipulator to avoid obstacles successfully. Theoretical results are presented for the proposed NTOA scheme (6) [as well as the noise-polluted NTOA scheme (7)] to show its excellent performance in motion planning and its remarkable noise suppression capability. On the basis of the PA10 robot manipulator with point and window-shaped obstacles and different kinds of noise, simulation results are provided to further substantiate the efficacy and superiority of the proposed NTOA scheme (6).

## Author contributions

DG had the basic ideas and made lots of research on neural dynamics and its application to redundant robot manipulators. FX made detailed scheme simulations and summarized the research results in the manuscript. LY, ZN, and HS revised the manuscript duly and carefully. All of the authors discussed the results at length.

### Conflict of interest statement

The authors declare that the research was conducted in the absence of any commercial or financial relationships that could be construed as a potential conflict of interest.
