# Supplementary material for: A New Noise-Tolerant Obstacle Avoidance Scheme for Motion Planning of Redundant Robot Manipulators
Source: Front Neurorobot. 2018 Aug 29;12:51. doi: 10.3389/fnbot.2018.00051 (PMC6124349; doi:10.3389/fnbot.2018.00051)
Supplement: Supplementary file 1 [file Data_Sheet_1.pdf]

Appendix A: Proof of Theorem 1

Based on the analysis in Section 2.2, the proposed NTOA scheme (6) can be reformulated as

$$J\dot{\theta} = \dot{r}_{\mathrm{d}} - k_{\mathrm{P}}(f(\theta) - r_{\mathrm{d}}) - k_{\mathrm{I}} \int_0^t (f(\theta) - r_{\mathrm{d}})\mathrm{d}\tau.$$

which is equivalent to

$$\dot{e}(t) = -k_{\mathrm{P}} e(t) - k_{\mathrm{I}} \int_0^t e(\tau) d\tau,$$

where $e(t) = f(\theta) - r_{\mathrm{d}}$ and $\dot{e}(t) = J\dot{\theta} - \dot{r}_{\mathrm{d}}$ with $J = \partial f(\theta)/\partial\theta$. (8) is a compact vector form of the following set of m decoupled equations:

$$\dot{e}_i(t) = -k_{\mathrm{P}} e_i(t) - k_{\mathrm{I}} \int_0^t e_i(\tau)\mathrm{d}\tau,$$

in which $i = 1, \cdots, m$. To analyze the ith subsystem (9), the following Lyapunov function candidate [38, 39] is defined:

$$v_i(t) = e_i^2(t) + k_{\mathrm{I}} \left(\int_0^t e_i(\tau)\mathrm{d}\tau \right)^2.$$

Evidently, $v_i(t) > 0$ for any $e_i(t) \neq 0$ or $\int_0^t e_i(\tau)\mathrm{d}\tau \neq 0$, and $v_{ij}(t) = 0$ only for $e_{ij}(t) = \int_0^t e_{ij}(\tau)\mathrm{d}\tau = 0$. This guarantees the positive-definiteness of the Lyapunov function candidate $v_i(t)$. For this Lyapunov function candidate, the time derivative $\dot{v}_i(t)$ can be derived as

$$\dot{v}_i(t) = \frac{\mathrm{d}v_i(t)}{\mathrm{d}t} = 2e_i(t)\dot{e}_i(t) + 2k_{\mathrm{I}} e_i(t) \int_0^t e_i(\tau)\mathrm{d}\tau = -2k_{\mathrm{P}} e_i^2(t) \leqslant 0,$$

which guarantees the negative-definiteness of $\dot{v}_i(t)$. On the basis of the Lyapunov theory, the trajectory of the Cartesian error $e(t)$ for the proposed NTOA scheme (6) can be concluded to be asymptotically stable. The proof is thus completed.

Appendix B: Proof of Theorem 2

Let us define $\varepsilon(t) = \int_0^t e(\tau)\mathrm{d}\tau \in R^m$. Then, $\dot{\varepsilon}(t) = e(t)$ and $\ddot{\varepsilon}(t) = \dot{e}(t)$. The ith subsystem (9) is thus rewritten as follows:

$$\ddot{\varepsilon}_i(t) = -k_{\mathrm{P}}\dot{\varepsilon}_i(t) - k_{\mathrm{I}}\varepsilon_i(t),$$

where $\varepsilon_i(t)$, $\dot{\varepsilon}_i(t)$, and $\ddot{\varepsilon}_i(t)$ are the ith elements of $\varepsilon(t)$, $\dot{\varepsilon}(t)$, and $\ddot{\varepsilon}(t)$, respectively.

As defined previously, the design parameters k_{P} and k_{I} satisfy $k_{\mathrm{P}}^2 > 4k_{\mathrm{I}}$ numerically. Therefore, let $\vartheta_1 = (-k_{\mathrm{P}} + \sqrt{k_{\mathrm{P}}^2 - 4k_{\mathrm{I}}})/2$ and $\vartheta_2 = (-k_{\mathrm{P}} - \sqrt{k_{\mathrm{P}}^2 - 4k_{\mathrm{I}}})/2$. Then, considering that $\varepsilon_i(0) = 0$ and $\dot{\varepsilon}_i(0) = e_i(0)$, the analytical solution to (10) is formulated as

$$\varepsilon_i(t) = \frac{e_i(0)(\exp(\vartheta_1 t) - \exp(\vartheta_2 t))}{\sqrt{k_{\mathrm{P}}^2 - 4k_{\mathrm{I}}}}.$$

Because $e(t) = \dot{\varepsilon}(t)$, the following result is further obtained:

$$e_i(t) = \frac{e_i(0)(\vartheta_1 \exp(\vartheta_1 t) - \vartheta_2 \exp(\vartheta_2 t))}{\sqrt{k_{\mathrm{P}}^2 - 4k_{\mathrm{I}}}}.$$

The vector-form Cartesian error $e(t)$ is thus derived as follows:

$$e(t) = \frac{e(0)(\vartheta_1 \exp(\vartheta_1 t) - \vartheta_2 \exp(\vartheta_2 t))}{\sqrt{k_{\mathrm{P}}^2 - 4k_{\mathrm{I}}}}.$$

On the basis of the above analysis (as well as the proof of Theorem 1), starting from an initial state $e(0) \neq 0$ (i.e., a nonzero initial error), the trajectory of the Cartesian error $e(t)$ for the proposed NTOA scheme (6) can be concluded to converge to zero exponentially. In other words, the Cartesian error $e(t)$ synthesized by (6) has the property of exponential convergence, which now completes the proof.

Appendix C: Proof of Theorem 3

Based on Appendix A, the noise-polluted NTOA scheme (7) can be reformulated as follows:

$$J\dot{\theta} = \dot{r}_{\rm d} - k_{\rm P}(f(\theta) - r_{\rm d}) - k_{\rm I}\int_0^t (f(\theta) - r_{\rm d})\mathrm{d}\tau + \delta(t),$$

which is equivalent to

$$\dot{e}(t) = -k_{\rm P}e(t) - k_{\rm I}\int_0^t e(\tau)d\tau + \delta(t).$$

By using the Laplace transformation [41], the ith subsystem of (11) is as follows:

$$se_i(s) - e_i(0) = -k_{\rm P}e_i(s) - \frac{k_{\rm I}}{s}e_i(s) + \delta_i(s).$$

Then, the following result is obtained:

$$e_i(s) = \frac{s(e_i(0) + \delta_i(s))}{s^2 + k_{\rm P}s + k_{\rm i}}.$$

Evidently, the transfer function is derived as follows:

$$\frac{s}{s^2 + k_{\rm P}s + k_{\rm I}},$$

of which the poles are $(-k_{\rm P} + \sqrt{k_{\rm P}^2 - 4k_{\rm I}})/2$ and $(-k_{\rm P} - \sqrt{k_{\rm P}^2 - 4k_{\rm I}})/2$. Note that, as defined previously, $k_{\rm P}$ and $k_{\rm I}$ satisfy $k_{\rm P}^2 > 4k_{\rm I} > 0$ numerically. Therefore, such two poles are located on the left half-plane, indicating the stability of the system (12).

Note that $\delta(t) = c \in R^m$ (being a constant), and thus $\delta_i(s) = c/s$. For (12), using the final value theorem [41] yields

$$\lim_{t\to\infty} e_i(t) = \lim_{s\to 0} se_i(s) = \lim_{s\to 0}\frac{s^2(e_i(0) + c/s)}{s^2 + k_{\rm P}s + k_{\rm I}} = 0.$$

According to (13), $\lim_{t\to\infty}\|e(t)\|_2 = 0$ with symbol $\|\cdot\|_2$ denoting the two norm of a vector. On the basis of the above analysis, the trajectory of $e(t)$ for the noise-polluted NTOA scheme (7) can be concluded to converge to zero. In other words, the Cartesian error $e(t)$ synthesized by (7) has a convergence property, which now completes the proof.

Appendix D: Proof of Theorem 4

As presented in Appendix C, the noise-polluted NTOA scheme (7) can be rewritten as follows:

$$\dot{e}(t) = -k_{\rm P}e(t) - k_{\rm I}\int_0^t e(\tau)d\tau + \delta(t),$$

where the ith ($\forall i = 1, \cdots, m$) subsystem is formulated as

$$\dot{e}_i(t) = -k_{\rm P}e_i(t) - k_{\rm I}\int_0^t e_i(\tau)d\tau + \delta_i(t).$$

Considering that $k_{\rm P}$ and $k_{\rm I}$ satisfy $k_{\rm P}^2 > 4k_{\rm I} > 0$ numerically, the solution to (14) is obtained as follows:

$$\begin{aligned} e_i(t) =& \frac{e_i(0)(\vartheta_1\exp(\vartheta_1 t) - \vartheta_2\exp(\vartheta_2 t))}{\sqrt{k_{\rm P}^2 - 4k_{\rm I}}} + \frac{1}{\sqrt{k_{\rm P}^2 - 4k_{\rm I}}}\int_0^t (\vartheta_1\exp(\vartheta_1(t-\tau)) \\ &- \vartheta_2\exp(\vartheta_2(t-\tau)))\delta_i(\tau)\mathrm{d}\tau. \end{aligned}$$

From the triangle inequality,

$$\begin{aligned} |e_i(t)| \leqslant& \frac{|e_i(0)(\vartheta_1\exp(\vartheta_1 t) - \vartheta_2\exp(\vartheta_2 t))|}{\sqrt{k_{\rm P}^2 - 4k_{\rm I}}} + \frac{\int_0^t |\vartheta_1\exp(\vartheta_1(t-\tau))||\delta_i(\tau)|\mathrm{d}\tau}{\sqrt{k_{\rm P}^2 - 4k_{\rm I}}} \\ &+ \frac{\int_0^t |\vartheta_2\exp(\vartheta_2(t-\tau))||\delta_i(\tau)|\mathrm{d}\tau}{\sqrt{k_{\rm P}^2 - 4k_{\rm I}}}. \end{aligned}$$

It follows the above inequality that

$$|e_i(t)| \leqslant \frac{|e_i(0)(\vartheta_1\exp(\vartheta_1 t) - \vartheta_2\exp(\vartheta_2 t))|}{\sqrt{k_{\rm P}^2 - 4k_{\rm I}}} + \frac{2}{\sqrt{k_{\rm P}^2 - 4k_{\rm I}}}\max_{0\leqslant\tau\leqslant t}|\delta_i(\tau)|.$$

Therefore, the following result is obtained:

$$\lim_{t\to\infty}\sup\|e(t)\|_2 \leqslant 2\sqrt{\frac{m}{k_{\rm P}^2 - 4k_{\rm I}}}\max_{0\leqslant\tau\leqslant t}|\delta_i(\tau)|.$$

On the basis of the above analysis, the Cartesian error $e(t)$ synthesized by the noise-polluted NTOA scheme (7) in the presence of bounded time-varying noise can be concluded to be bounded, with the steady-state error being bounded by $2\sqrt{m}\max_{0\leqslant\tau\leqslant t}|\delta_i(\tau)|/\sqrt{k_{\rm P}^2 - 4k_{\rm I}}$. The proof is thus completed.
